# A novel p.E276K *IDUA* mutation decreasing α**-**L**-**iduronidase activity causes mucopolysaccharidosis type I

**Published:** 2011-02-11

**Authors:** Korrakot Prommajan, Surasawadee Ausavarat, Chalurmpon Srichomthong, Vilavun Puangsricharern, Kanya Suphapeetiporn, Vorasuk Shotelersuk

**Affiliations:** 1 Center of Excellence for Medical Genetics, Department of Pediatrics, Faculty of Medicine, Chulalongkorn University, Bangkok, Thailand; 2 Molecular Genetics Diagnostic Center, King Chulalongkorn Memorial Hospital, Thai Red Cross, Bangkok, Thailand; 3 Department of Ophthalmology, Faculty of Medicine, Chulalongkorn University, Bangkok, Thailand

## Abstract

**Purpose:**

To characterize the pathogenic mutations causing mucopolysaccharidosis type I (MPS I) in two Thai patients: one with Hurler syndrome (MPS IH), the most severe form, and the other with Scheie syndrome (MPS IS), the mildest. Both presented with distinctive phenotype including corneal clouding.

**Methods:**

The entire coding regions of the α-L-iduronidase (*IDUA*) gene were amplified by PCR and sequenced. Functional characterization of the mutant *IDUA* was determined by transient transfection of the construct into COS-7 cells.

**Results:**

Mutation analyses revealed that the MPS IH patient was homozygous for a previously reported mutation, c.252insC, while the MPS IS patient was found to harbor a novel c.826G>A (p.E276K) mutation. The novel p.E276K mutation was not detected in 100 unaffected ethnic-matched control chromosomes. In addition, the glutamic acid residue at codon 276 was located at a well conserved residue. Transient transfection of the p.E276K construct revealed a significant reduction of IDUA activity compared to that of the wild-type IDUA suggesting it as a disease-causing mutation.

**Conclusions:**

This study reports a novel mutation, expanding the mutational spectrum for MPS I.

## Introduction

Mucopolysaccharidoses (MPs) are a group of inherited lysosomal storage disorders resulting from a deficiency of enzymes that catalyze the degradation of glycosaminoglycans (GAGs). MPS I is considered as the prototypic lysosomal storage disease of the MPS group which is caused by a deficiency of lysosomal α-L-iduronidase (IDUA, EC 3.2.1.76). As a result of defects inside the lysosomes, partially degraded GAGs, heparan, and dermatan sulfate accumulate in these organelles leading to progressive cellular dysfunction and characteristic features of the disorder. MPS I has been classified into three clinical phenotypes, with different levels of severity: a severe form (Hurler syndrome; MPS IH; OMIM 607014), an intermediate form (Hurler-Scheie syndrome; MPS IH/S; OMIM 607015), and a mild form (Scheie syndrome; MPS IS; OMIM 607016) [1,2].

MPS I can be diagnosed biochemically by the presence of urinary dermatan sulfate and heparan sulfate and the significant reduction or absence of IDUA activity in patients’ leukocytes or skin fibroblasts [1]. The *IDUA* gene contains 14 exons encoding a 653-amino acid precursor protein [3,4]. At least 110 different disease-causing mutations in *IDUA* have been described with the majority being missense/nonsense mutations. The splice-junction alterations and nucleotide insertions/deletions have also been reported (Human Gene Mutation Database, accessed September, 2010). The p.W402X and p.Q70X mutations are most commonly found in Caucasians and are accountable for as much as 70% of the disease alleles in some European countries [5-7].

There has been only one report on Thai patients with molecularly confirmed Hurler syndrome [8]. Here, we described two unrelated Thai patients with MPS I and identified one recurrent and one novel mutations in *IDUA*. The functional consequence of the novel missense mutation was also further elucidated.

## Methods

### Subjects

Two unrelated Thai patients with clinically diagnosed MPS I were reported. Patient 1, a product of a consanguineous marriage, with clinical features consistent with MPS IH was diagnosed at one year of age. The clinical findings included coarse facial features, corneal clouding, hepatosplenomegaly, and skeletal deformities. He was noted to have delayed development and passed away at age two from severe respiratory infection. Patient 2 was adopted and was diagnosed with MPS IS at the age of 29 years. The clinical features included coarse facial features, corneal clouding and claw hand deformity. The α-L-iduronidase activity in leukocytes from patient 2 was measured and revealed a significant reduction in enzyme activity (0.61 nmol/h/mg protein). The mean of α-L-iduronidase activity in leukocytes obtained from eight unaffected adult Thai controls was 23.10±8.80 nmol/h/mg protein. Unfortunately, leukocytes from patient 1 were unavailable for analysis.

### Mutation analysis of the *IDUA* gene

After informed consent, genomic DNA was extracted from peripheral blood leukocytes from patients and available parents according to standard protocols. The entire coding regions of *IDUA* were assessed by polymerase chain reaction (PCR) and direct sequencing. Most of the oligonucleotide primers were used as previously described [9] and presented in Table 1. Exons 3–6, 13–14, and their intron-exon boundaries were amplified using newly designed primers (Table 1). The PCR products were treated with ExoSAP-IT (USP Corporation, Cleveland, OH), according to the manufacturer’s recommendations, and sent for direct sequencing at the Macrogen, Inc. (Seoul, Korea). The sequences were analyzed using Sequencher (version 4.2; Gene Codes Corporation, Ann Arbor, MI).

**Table 1:** Primers and PCR conditions for *IDUA* mutation analysis.

**Exon**	**Primer name**	**Primer sequences for PCR (5′ to 3′)**	**Product size (bp)**	**Melting temperature (°C)**
1	IDUA-Ex1F	F-ACCCAACCCCTCCCAC	398	58
	IDUA-Ex1R	R-AGCTTCAGAGACCGGAG		
2	IDUA-Ex2F	F-GAACGTGTGTGTCAGCCG	304	62
	IDUA-Ex2R	R-GCTCGGAAGACCCCTTGT		
3–4	IDUA-Ex3/4F	F-TTCCAGCCTGGAGCATGGAG	516	62
	IDUA-Ex3/4R	R-GTTGCACCCCTATGACGCAG		
5–6	IDUA-Ex5/6F	F-TCACCTTGCACCCTCCCTCC	576	62
	IDUA-Ex5/6R	R-GCTGACCCTGGTGGTGCTGA		
7	IDUA-Ex7F	F-TGCGGCTGGACTACATCTC	448	62
	IDUA-Ex7R	R-GCAGCATCAGAACCTGCTACT		
8	IDUA-Ex8F	F-CCACCTTCCTCCCGAGAC	386	62
	IDUA-Ex8R	R-GGAGCGCACTTCCTCCAG		
9–10	IDUA-Ex9F	F-TCCTTCACCAAGGGGAGG	701	58
	IDUA-Ex10R	R-TCCTCAGGGTTCTCCAGG		
11–12	IDUA-Ex11/12F	F-GTGTGGGTGGGAGGTGGA	466	62
	IDUA-Ex11/12R	R-CTTCACCCATGCGGTCAC		
13–14	IDUA-Ex13/14F	F-CTGCCTGCTCCCACCTTTGHA	530	62
	IDUA-Ex13/14R	R-CCCATGCTGCCCTCCCATCA		

For a novel missense mutation, c.826G>A (p.E276K), PCR-RFLP analysis with the MboII restriction enzyme was used to confirm its presence in the patient and to screen in 100 ethnic-matched unaffected control chromosomes. The forward and reverse primers for amplification of *IDUA* exon 7 were used to generate a 448-bp PCR product. The mutant PCR product creates another MboII site, which allowed detection of the mutation by agarose gel electrophoresis. As the proband was adopted, the parental DNA was not available for analysis.

### Protein sequence comparison

IDUA orthologs were first identified through a BLAST search of the non-redundant database using *Homo sapiens* IDUA, accession NP_000194.2 as the reference sequence. All known and complete IDUA sequences were included from the vertebrate lineage. These files in FASTA format were then analyzed by ClustalX program.version 2.0.12. The human IDUA was aligned with rat (*Rattus norvegicus*; NP_001165555.1), mouse (*Mus musculus*; NP_032351.1), cow (*Bos taurus*; XP_877410.2), chicken (*Gallus gallus*; NP_001026604.1), frog (*Xenopus laevis*; NP_001087031.1), and zebrafish (*Danio rerio*; XP_001923689.1). The program classified amino acids by the variation in polarity, assessing both amino acid class conservation and evolutionary conservation at any given site.

### Construction of plasmids and site*-*directed mutagenesis

The expression vector wild-type pEFNeo-IDUA was kindly provided by Hopwood’s laboratory. The mutant *IDUA* constructs of c.1206G>A (p.W402X) and c.826G>A (p.E276K) were generated by in vitro site-directed mutagenesis (QuickChange site-directed mutagenesis kit; Stratagene, La Jolla, CA) on the pEFNeo-IDUA using oligonucleotide primers. The p.W402X was a previously described mutation in MPS IH and was used as a mutant control. All mutant *IDUA* constructs were verified by direct sequencing.

### Transient transfection and enzyme assay

COS-7 cells were grown in Dulbecco’s Modified Eagle Medium supplemented with 10% fetal bovine serum at 37 °C and 5% CO_2_. COS-7 cells were transfected with the wild- type or mutant constructs using Lipofectamine™ 2000 (Invitrogen, Carlsbad, CA), according to the manufacturer’s instructions. Cells were harvested after 48 h and assayed for IDUA activity. Experiments were performed twice with triplicate per experiment.

An assay for IDUA activity was performed using the fluorogenic substrate 4-methylumbellliferyl-α-L-iduronide (Glycosynth, Cheshire, UK) as previously described [10]. The protein concentration of the cell lysates was determined by the Bradford assay.

## Results

**Figure 1:**
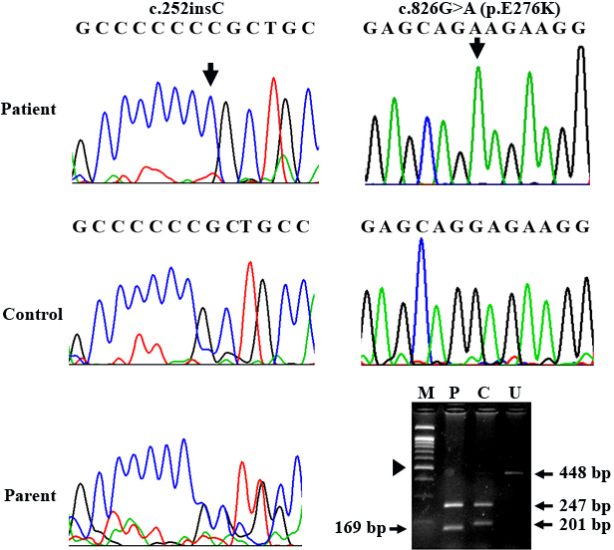
Mutation analysis. The left and right panels relate to c.252insC and c.826G>A (p.E276K) mutations, respectively. Upper, middle and left lower panels are electropherograms of patients, unaffected controls, and one of the parents, respectively. Each identified mutation is indicated by an arrow. Right lower panel showing RFLP analysis for the c.826G>A mutation in patient 2. MboII digested the wild-type allele of a control into 247 and 201-bp products. The c.826G>A creates another cleavage site for MboII resulting in 169 and 32-bp bands. Note that the 32-bp band is not visualized. (M=100-bp marker, P=patient, C=control, U=uncut amplified product). The 500-bp band is indicated by an arrowhead.

PCR-sequencing revealed a homozygous c.252insC mutation in patient 1 (Figure 1, left upper panel). Sequence analysis of the parental genomic DNA confirmed that both parents were heterozygous for the c.252insC. This mutation has been previously described to be associated with a severe phenotype in different populations including Thai [6,8].

A novel homozygous c.826G>A mutation in exon 7 was identified in patient 2. This was expected to result in a glutamic acid to lysine substitution (p.E276K) at codon 276 (Figure 1, right upper panel). No other variants were observed.

**Figure 2:**
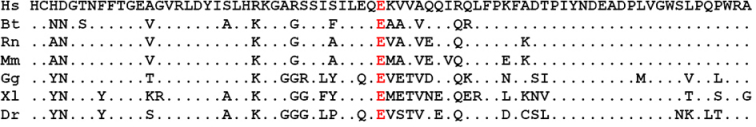
Protein sequence alignment of IDUA from different species. The site of the amino acid variant found in this study is indicated in bold red in all conserved species. Sites that are 100% conserved across all sequences are indicated by dots (.). Hs, *Homo sapiens*; Bt, *Bos taurus*; Rn, *Rattus norvegicus*; Mm, *Mus musculus*; Gg, *Gallus gallus*; X1, *Xenopus laevis*; Dr, *Danio rerio*.

Restriction enzyme digestion of the PCR products with MboII was used to confirm the presence of the c.826G>A mutation in the patient 2 and its absence in 100 ethnic-matched unaffected control chromosomes. Alignment of the IDUA protein sequences revealed that the glutamic acid residue at codon 276 was located at a well conserved residue (Figure 2).

The functional effect of the novel p.E276K and the previously identified p.W402X mutations on IDUA activity were analyzed by transient transfection of each IDUA construct into COS-7 cells. Comparing to the enzyme activity of the wild-type IDUA (435.04±56.23 nmol/h/mg protein), the p.E276K and the p.W402X had reduced activity to background levels (31.88±6.05 nmol/h/mg protein and 21.10±12.57 nmol/h/mg protein, respectively, Table 2).

**Table 2:** α-L-iduronidase activity in transiently transfected COS-7 cells with either wild-type or mutant IDUA constructs.

**Constructs**	**α-L-iduronidase activity (nmol/h/mg cell protein) mean±SD**	**Phenotype**
None	27.17±4.89	-
pEFNeo	32.52±10.58	-
pEFNeo/IDUA	435.04±56.23	-
pEFNeo/p.W402X	21.10±12.57	Hurler
pEFNeo/p.E276K	31.88±6.05	Scheie

## Discussion

We identified two unrelated Thai patients with MPS I. Patient 1 was found to be homozygous for the c.252insC mutation. It has been previously described to be responsible for a severe phenotype. The novel missense mutation, c.826G>A (p.E276K), was identified in patient 2 with MPS IS and caused a significant reduction of IDUA activity.

There was only one report describing two patients with MPS IH in the Thai population. Four different mutations including the c.252insC were detected. Combined this previous report with our findings, the c.252insC mutation is responsible for 40% (2 out of 5 alleles), making it a possible common mutant allele in Thai patients with MPS IH. Continued studies for mutations in patients with MPS I will be required for a definite conclusion. If this is proved to be the case, it will benefit a molecular diagnosis for this population.

Our patient with MPS IS was found to carry the novel c.826G>A (p.E276K) mutation. Due to the unavailability of her parent’s DNA, a possibility that the other allele is deleted making her hemizygous for the c.826G>A remains. As no other variants were observed, the patient could be either homozygous or hemizygous for this particular mutation.

Several lines of evidence support the pathogenicity of this novel mutation. First, it is not found in 100 ethnic-matched control chromosomes. Second, the glutamic acid at codon 276 is located at a well conserved residue (Figure 2). And most importantly, when transiently transfected COS-7 cells with the p.E276K construct, IDUA enzyme activity was reduced to background levels. We also tested for the effect of the p.W402X, one of the most common mutations found in Caucasian individuals with MPS I, and revealed a significant reduction of IDUA activity similar to previous reports [9]. Our studies therefore suggested the p.E276K as a disease-causing mutation.

Prediction of a patient’s clinical phenotype through genetic analysis of *IDUA* has been complicated by the high number of disease-causing mutations and polymorphic variants present in the *IDUA* gene. It has been hypothesized that a combination of mutations, polymorphisms, genetic background, and environmental factors contribute to the clinical phenotypic spectrum [9,11,12]. Even though studies of genotype-phenotype correlations for MPS I patients are sometimes inconclusive, the nonsense mutations if present on both *IDUA* alleles have been shown to cause a severe form of MPS I. The c.252insC causing frameshift identified in our patient with MPS IH has been previously reported in two patients, one from the Netherlands and the other from Thailand [6,8]. Both were compound heterozygotes for the c.252insC and other nonsense mutations. Our finding of another MPS IH patient associated with the homozygous c.252insC mutation further supports the deleterious effect of this particular mutation on the enzyme activity.

In summary, we reported two unrelated Thai patients with MPS I with different clinical severity who were found to carry different mutations. Combined with a previous study in Thai patients, the c.252insC mutation identified in our patient with MPS IH, could be a common mutation causing Hurler syndrome in the Thai population. We also detected a novel missense mutation of the *IDUA* gene, c.826G>A (p.E276K) causing Scheie syndrome.

Transient transfection studies and assay for IDUA activity confirmed its pathogenic role. This study expands the mutational spectrum of MPS I.
